# Case Report: Sequential Liver After Kidney Transplantation in a Patient With Sensenbrenner Syndrome (Cranioectodermal Dysplasia)

**DOI:** 10.3389/fped.2022.834064

**Published:** 2022-02-25

**Authors:** Joanna Ryżko, Joanna Walczak-Sztulpa, Piotr Czubkowski, Anna Latos-Bieleńska, Adam Kowalski, Marek Stefanowicz, Wioletta Jarmużek, Ryszard Grenda, Joanna Pawłowska

**Affiliations:** ^1^Department of Gastroenterology, Hepatology, Nutritional Disorders and Pediatrics, The Children's Memorial Health Institute, Warsaw, Poland; ^2^Department of Medical Genetics, Poznan University of Medical Sciences, Poznan, Poland; ^3^Department of Pediatric Surgery and Organ Transplantation, The Children's Memorial Health Institute, Warsaw, Poland; ^4^Department of Nephrology, Kidney Transplantation and Hypertension, The Children's Memorial Health Institute, Warsaw, Poland

**Keywords:** ciliopathy, Sensenbrenner syndrome (cranioectodermal dysplasia), renal failure, liver disease, kidney and liver transplantation

## Abstract

Sensenbrenner syndrome, also known as cranioectodermal dysplasia (CED), is a rare ciliopathy clinically characterized by congenital craniofacial, skeletal, and ectodermal defects. Chronic kidney and liver insufficiency are also present in this disorder. Cranioectodermal dysplasia is an autosomal recessive and heterogeneous genetic disease. Six genes (IFT122, WDR35, IFT140, IFT43, IFT52, and WDR19) are known to be associated with this syndrome. Until 2021 more than 70 patients have been reported with CED, however, an orthotopic liver transplantation has been reported only in one case. Here, we present a case report of sequential liver-after-kidney transplantation in a male patient affected by CED. The kidney and liver transplantation was performed at the age of 7 and 12 years, respectively. Patients with Sensenbrenner syndrome require a multidisciplinary medical management and should regularly be followed-up by hepatologists and nephrologists, as the liver and kidney diseases are the major cause of morbidity and mortality.

## Introduction

Sensenbrenner syndrome, also known as cranioectodermal dysplasia (CED), is a rare and heterogeneous ciliopathy inherited in an autosomal recessive manner.

The characteristic clinical features include: craniosynostosis, dolichocephaly, facial dysmorphisms, growth retardation, short limbs, narrow chest, chronic kidney, and liver disease. To date, more than 70 patients with CED have been described and variants in six genes have been linked to this syndrome: IFT122, WDR35, IFT140, IFT43, IFT52, and WDR19 (1, 2). Changes in IFT122 and WDR35 account for approximately 70% of the incidence of this syndrome. Products of genes associated with CED belong to the intraflagellar transport (IFT) process, which plays an essential role in a proper cilia formation and function. Cilia are present in most types of human cells, including those localized in the kidneys, liver, eyes, heart, and limbs. Moreover, the cilia regulate diverse signaling pathways, such as the hedgehog (Hh) and Wnt-signaling (Wnt), which play a crucial role in development and postnatal maintenance of organs and tissues. Therefore, ciliopathies are complex and multisystem diseases which involve all the major organs (3–5).

The main underlying cause of liver disease in ciliopathies is related to abnormal development of the bile ducts, which is often manifested as a congenital hepatic fibrosis (CHF), as well as Caroli's disease (CD) or polycystic liver disease (PLD) (6, 7).

In CED, hepatic lesions of a variable severity have been observed, ranging from hepatosplenomegaly, an increased echogenicity of the liver or a presence of liver cysts in imaging evaluations, to the severe liver failure with hyperbilirubinemia or cholestasis, requiring hospitalization as early as in the neonatal period. In contrast, a cirrhosis with severe cholestasis and biliary proliferation, as well as an acute cholangitis, has been described in infants, while in children aged 10 months, 3, and 4 years the presence of liver cysts have been confirmed (8, 9). Moreover, fatal outcome of two patients with CED at the age of 4 months, due to kidney and liver failure has been reported (1).

The intrahepatic biliary dilatation, biliary proliferation, and fibrosis were confirmed in the histopathological examination of the liver samples taken from the CED patients. Moreover, the elevated activity of hepatic enzymes may be also present (2). Affected individuals with kidney failure (KF), and end-stage liver disease (ESLD) may be the candidates for organ transplantation procedures, which are regarded as a life-saving management in relevant cases. The differential diagnosis of CED with Caroli's disease/syndrome and ARPKD is presented in Table 1.

**Table 1:** Differential diagnostics CED with Caroli's disease/syndrome and ARPKD.

	**Craniectodermal dysplasia**	**Caroli's disease/syndrome**	**ARPKD**
Liver	• Hepatosplenomegaly • Liver cirrhosis • Acute cholangitis • Liver cyst • Histopathological examination—fibrosis, defective remodeling of the ductal plate (“DMP-like”), cholestasis	• Cholangitis • Hepatolithiasis • Gallblader stones • May present with CHF (Caroli's syndrome) with progressive development of portal hypertension • USG and CT studies may visualize liver cyst and possible intrahepatic lithiasis and provide information on the common bile duct • MR imaging can visualize small cysts (2 mm in diameter) • Histopathological examination in Caroli's disease—intrahepatic bile duct extasie and proliferation are associated with severe periportal fibrosis • Histopathology examinations in CHF—abnormal portal tracts with an excess number of abnormally shaped embryonic bile ducts retained in their primitive ductal plate configuration, an abnormal portal vein, and periportal fibrosis without inflammation	• Liver lesions may not be visible at 1 year of age • Hepatosplenomegaly • Hepatomegaly and symptoms of portal hypertension usually seen in older children • The liver is always involved, but in the neonatal and infancy the picture of kidney disease predominates • Histopathological examination—ductal plate malformation
Kidney	• Kidney failure (usually occurs between 2 and 6 years of age) • Hypertension, proteinuria/hematuria • Histopathological examination—interstitial fibrosis with focal inflammatory cell infiltrates, tubular atrophy, glomerulosclerosis, occasional cysts	• Renal cystic disease • Medullary sponge kidney	• Most present perinatally with enlarged kidneys • Systemic hypertensions • Cysts 1–2 mm in diameter arise due to dilatation of the collecting tubules of the nephron—may be poorly visible on ultrasound examination

*CED, cranioectodermal dysplasia; ARPKD, autosomal recessive polycystic kidney diseases; CHF, congenital hepatic fibrosis*.

## Case Report

Here, we present a 16-year-old male patient diagnosed with CED.

The patient is a second child of the otherwise healthy, however consanguineous parents. He was delivered after uncomplicated pregnancy at 38 week of gestation. His birth weight was 3.54 kg (>50^th^ percentile), birth length of 56 cm (>98^th^ percentile), and occipital frontal circumference (OFC) 40.5 cm (>98^th^ percentile). Apgar score was 7 at 1 min.

**Figure 1:**
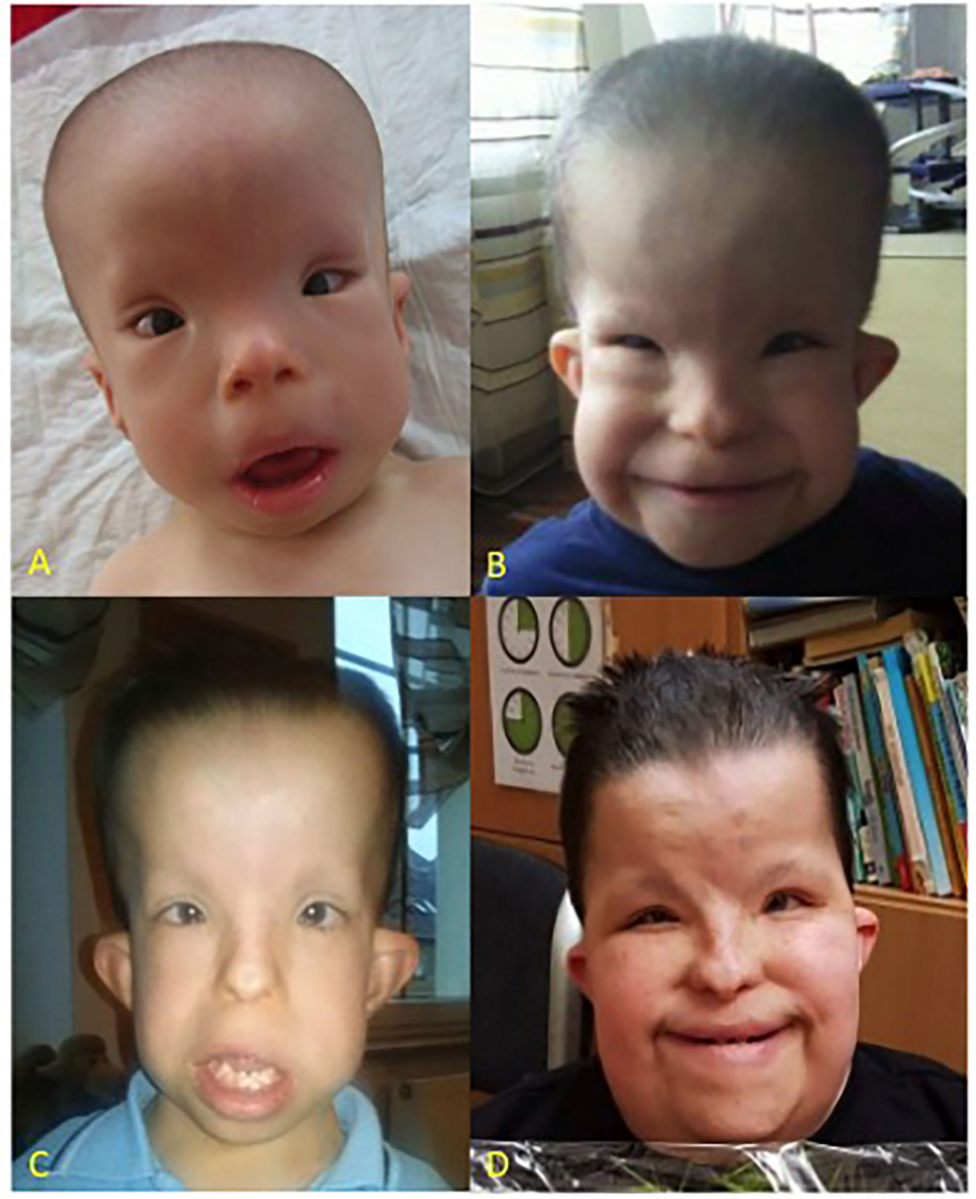
Patient at the age of 1 year **(A)**, 5 years **(B)**, 9 years **(C)**, and 16 years **(D)**, respectively. CED characteristic dysmorphic features include dolichocephaly, high forehead, epicanthus, telecanthus, broad nasal bridge, hypertelorism, full cheeks, low set ears.

A clinical examination performed directly after birth, revealed the limb shortening, a narrow thorax, brachydactyly, large bilateral inguinal hernias, and dysmorphic features such as dolichocephaly, highly protruding auricles, small flat nose, and hypertelorism (Figure 1).

The clinical features observed in this patient were very similar to those presented by his older sister, who has also been diagnosed with Sensenbrenner syndrome.

A genetic analysis of the family identified a missense variant p.Val553Gly (c.1658T>G) in the IFT122 gene as previously described by Walczak-Sztulpa et al. (1). This change was the first variant found in the IFT122 gene in the family with Sensenbrenner syndrome. Based on the research project this gene has been shown to be associated with CED. p.Val553Gly change is consistent with the CED phenotype as both parents are the heterozygous carriers and two affected children are homozygous for the identified variant. The variant has been classified as likely pathogenic according to the ACMG guidelines (Varsome tool, the 16^th^ January 2022) (10).

Functional studies performed in the skin fibroblasts harvested from the male patient revealed a significantly reduced cilia frequency and length, as compared to controls confirming abnormal primary cilia morphology and formation in the affected individual (1).

In infancy, the patient presented elevated creatinine serum concentration and later, over the following month a regular deterioration of the kidney function was observed. Finally he developed the KF at the age of 3.5 years, which required a chronic dialysis program. Consequently, he underwent a deceased-donor kidney transplantation at the age of 7 years and remained on a triple maintenance immunosuppression (steroids, tacrolimus, mycophenolic acid). At that time, the splenomegaly was diagnosed, however without significant complications of portal hypertension and synthetic liver function was preserved. Later he developed the esophageal varices requiring ligation, at 3 years after a kidney transplantation.

At the age of 12 years, due to, hepatic decompensation (increasing ascites), the patient was hospitalized in the local hospital. Laboratory tests showed elevated concentration of urea and creatinine, hypoproteinemia and hypoalbuminemia, abnormal coagulogram, anemia, and thrombocytopenia.

An abdominal ultrasound examination showed a large volume of fluid in the abdominal cavity, a reduced liver size with irregular outlines and features of the fibrosis and an enlarged spleen sized 14 × 8 cm (reference range: 8–9 cm). During hospitalization, the patient received red cell, platelets and albumin transfusions. A paracentesis was performed, yielding a total of approximately 4 L of ascitic fluid. Then the patient was transferred to our hospital. On physical examination, he presented a large abdominal circumference (approximately 100 cm), a translucent vascular network on the abdomen (“caput medusae”) while the size of the liver and spleen could not be assessed. Laboratory tests showed: leukopenia (WBC 2.2 K/μl; reference range: 6–17.5) and thrombocytopenia (PLT 58 K/μl, reference range: 140–635) a mild anemia (RBC 3.9 M/μl, reference range: 3.8–5.2); Hb 10.6 g/dl (reference range: 10–13.2); Ht 39.4% (reference range: 33–39), slightly abnormal coagulogram [INR 1.64, APTT 31 (sek), Fg 1.08 g/l], hypoalbuminemia (36.3 g/l: reference range: 35–49) and hypoproteinemia (58.2 g.l, reference range: 65–81), electrolyte abnormalities (Ca 2.01 mmol/l, reference range: 2.3–4.6; P 0.94 mmol/l; reference range: 0.84–1.61; Mg 0.47 mmol/l, reference range: 0.7–1.05). Proteinuria (urinary protein 100 mg/dl) was found in the urinalysis. The calculated PELD score was 16.

An abdominal ultrasound revealed a large volume of fluid in the abdominal cavity, a small liver with polycyclic outlines and an increased echogenicity without focal changes. The spleen was significantly enlarged, presenting a size of 20 × 10 cm (reference range: 8–9). Paracentesis, treatment with diuretics and albumin transfusions were continued, resulting in the reduction of abdominal circumference to the values seen before decompensation. A follow-up ultrasound showed the presence of a trace of fluid in the abdominal cavity. Overall, the patient has lost about 8 kg of the body weight. During hospitalization a gastroscopy was performed, which showed wide bands of esophageal varices along the entire length of the esophagus. Banding was performed which resulted in a complete smoothing of the esophagus.

Due to the severity of portal hypertension, episode of ascites and hypersplenism, the patient was qualified for the liver transplantation. The liver from a deceased donor was transplanted, and directly prior to the liver transplantation, a splenectomy was performed.

**Figure 2:**
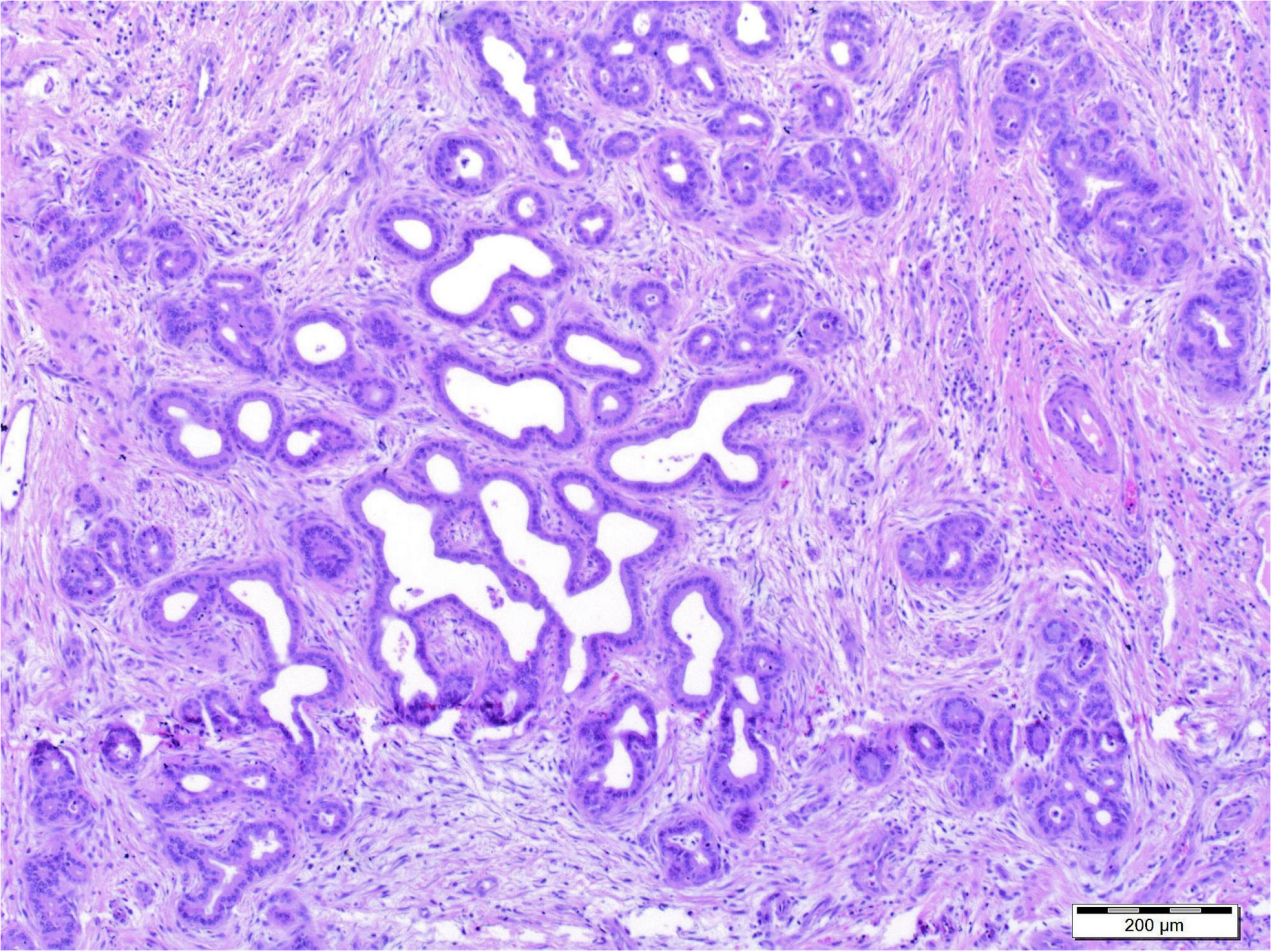
Histopathological examination of the removed liver showed fibrosis with features of cirrhosis and the presence of abnormal morphogenesis of intrahepatic bile ducts with “DPM-like” images. The lesions were accompanied by features of cholestasis, focal with severe intensity.

A histopathological examination of the removed liver showed fibrosis with features of cirrhosis and the presence of abnormal morphogenesis of intrahepatic bile ducts with “DPM-like” images. The lesions were accompanied by features of cholestasis, focal with severe intensity (Figure 2). In the perioperative period the patient required temporary dialysis, which was continued for 5 days as continuous veno-venous hemodiafiltration (CVVHDF). Currently, after 3 years, the patient's liver function is normal, while the function of the transplanted kidney is currently deteriorating, as eGFR decreased to 44 ml/min/1.73 m^2^ due to the ongoing chronic allograft nephropathy.

**Figure 3:**
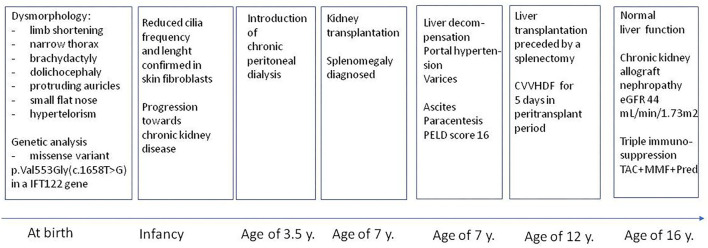
Clinical course during 16-year follow-up.

Outcome in terms of maintaining the kidney graft function is moderate in the range of forthcoming 5 years, as the retransplantation will be probably required due to the developed progressive chronic nephropathy in a primary graft, however it should not affect patient survival. Prognosis for proper liver function is excellent as far as he is compliance. Triple immunosuppression (TAC+MMF+Pred) is continued. The clinical course during the 16-year follow up is illustrated in Figure 3.

## Discussion

Sensenbrenner syndrome is a rare genetic syndrome. The incidence in the US has been reported for <1:1,000,000 live births. Usually symptoms manifest themselves in the first years of life (11). To date, 41 families with confirmed diagnosis of CED by genetic analysis have been reported in the literature (9, 12–17). The diagnosis of Sensenbrenner syndrome is made based on characteristic clinical features and molecular genetic testing, usually in the first years of life. In affected patients mortality might be associated with kidney and liver insufficiency, respiratory and heart failure, and hypovolemic shock (1, 9).

Liver involvement of some degree is known to occur in approximately 40–50% of patients with Sensenbrenner syndrome, regardless of mutation status, also severe liver involvement is a less common feature (8). In rare cases KF may develop as early as in an infant period, due to congenital tubulointerstitial nephropathy (18), however progression to severe KF rather takes more time (a couple of years) in patients presenting nephronophtysis (16, 19).

The reported patient presented ultrasonography picture suggesting chronic tubulointerstitial lesions in the native kidneys, requiring the introducing of regular haemodialysis at the age of 3.5 years. At the age of 7 he received a kidney transplant. At that time a liver function was normal, and only mild splenomegaly was confirmed. More severe pathology was presented at the age of 10 years, when he developed esophageal varices that required banding. Two years later, at the age of 12, a liver (after kidney) transplantation had to be performed due to the deteriorating liver function.

End-stage liver disease requiring transplantation has been reported just in one 7 year-old male patient with CED and WDR35 variants (Table 2). Deteriorating liver function with bleeding from esophageal varices, and deteriorating nutritional status was diagnosed in this case. Moreover, due to concomitant kidney insufficiency [stage 3 of chronic kidney disease (CKD)], a simultaneous liver and kidney transplantation was initially considered, but as the liver failure was more life-threatening, the transplantation procedure was limited to the isolated liver transplantation (11).

**Table 2:** Liver and kidney transplantation in Sensenbrenner syndrome.

	**Gene**	**Sex**	**Age at transplantation** **(years)**	**Reference (No.)**
**Liver**
1.	WDR35	Male	7	(10)
**Kidney**
1.	WDR35	Female	5	(20)
2.	WDR35	Male	4	(21)
3.	WDR35	Female	5	(22)
4.	WDR35	Male	2.5	(13)
5.	WDR35	Male	14	(14)
6.	WDR35	Female	7	(14)
7.	WDR35	Male	9	(11)
8.	IFT140	Male	6	(23)
9.	IFT140	Male	5	(24)
10.	IFT144/WDR19	Female	14	(19)
11.	IFT144/WDR19	Female	2	(25)
12.	IFT144/WDR19	Female	2	(14)

From the literature we also know that combined liver and kidney transplantation has also been proposed for one male CED individual with IFT122 compound heterozygous changes (26).

In the reported patient, a sequential organ transplantation was performed due to the fact that kidney function deteriorated quite early, while a relatively good liver function was maintained until the age of 10 years. Progressive liver fibrosis, which developed over several years finally led to the liver failure.

Chronic kidney disease is one of the common multiorgan features of Sensenbrenner syndrome, however with variable outcomes in terms of the degree of KF (at the moment of case publication). Several patients develop stage 5 (end-stage) of CKD and require kidney replacement therapy, including dialysis and (sequentially) a kidney transplantation. We have found 12 confirmed cases published in the last decade, in whom complete genetic diagnosis was available and the kidney transplantation was performed (Table 2) (nevertheless some other developed CKD 5 and are awaiting transplantation). The age at transplantation varied from 2.5 to 14 years. The genetic background included WDR35, IFT140, and IFT144/WDR19 genes mutations. The currently reported patient, who required sequential kidney and liver (after-kidney) transplantation presented IFT122 mutation. To our best knowledge, this is the first report of a sequential liver-after-kidney transplantation, successfully performed in a pediatric patient with Sensenbrenner syndrome.

## Conclusion

Patients with Sensenbrenner syndrome should be followed up by hepatologists and nephrologists on regular basis, as the liver and kidney diseases are the major causes of morbidity and mortality. The choice of transplantation procedure has to be individually assessed depending on the severity of kidney and liver damage over time.

## Data Availability Statement

The original contributions presented in the study are included in the article/supplementary material, further inquiries can be directed to the corresponding author/s.

## Ethics Statement

Written informed consent was obtained from the minor(s)' legal guardian/next of kin for the publication of any potentially identifiable images or data included in this article.

## Author Contributions

JR, JW-S, PC, AL-B, AK, MS, WJ, RG, and JP: contributed interpretation of the data and critical revision of the article. All authors contributed to the article and approved the submitted version.

## Conflict of Interest

The authors declare that the research was conducted in the absence of any commercial or financial relationships that could be construed as a potential conflict of interest.

## Publisher's Note

All claims expressed in this article are solely those of the authors and do not necessarily represent those of their affiliated organizations, or those of the publisher, the editors and the reviewers. Any product that may be evaluated in this article, or claim that may be made by its manufacturer, is not guaranteed or endorsed by the publisher.
